# Midwives’ willingness to provide home-based maternity and child health care, along with the associated factors and barriers, in Gondar, Ethiopia

**DOI:** 10.3389/fgwh.2025.1442897

**Published:** 2025-12-01

**Authors:** Alemneh Tadesse Kassie, Kindu Yinges Wondie, Tewodros Seyoum

**Affiliations:** 1 Department of Clinical Midwifery, School of Midwifery, College of Medicine & Health Sciences, University of Gondar, Gondar, Ethiopia; 2 Department of Women’s and Family Health, School of Midwifery, College of Medicine & Health Sciences, University of Gondar, Gondar, Ethiopia

**Keywords:** maternity, child health care, home-based care, willingness, Ethiopia, midwives’

## Abstract

**Introduction:**

Many Ethiopian mothers and their infants do not have access to maternity and child health care during the first week after birth, increasing their risk of becoming ill or dying. Midwives doing maternity and child health care home visits could improve the overall maternity and child health care experience. There is inadequate empirical evidence to show the desire of midwives to implement home-based maternity and child health care in low-income countries like Ethiopia.

**Methods:**

Between February 27 and October 27, 2023, 423 midwives participated in an institutional-based mixed methods, cross-sectional study. For the quantitative study, data were collected using a standardized self-administered questionnaire, whereas for the qualitative study, we conducted in-depth interviews with 12 midwives'. Bivariate and multivariable logistic regression analyses were performed. The adjusted odds ratio with confidence intervals at *P*-value 0.05 was used to identify a statistically significant relationship between the independent and outcome variables. Thematic analysis was used to interpret qualitative data.

**Result:**

The percentage of midwives' who would be willing to offer child health care and home-based maternity care was 74.3%, with a 95% confidence interval between 74% and 77.4%. A strong correlation was found between midwives' high level of willingness to Implement home-based maternity and child health care and their history of obstetrics-related family loss (aOR: 2.2 with CI (1.04–4.8), *P* = 0.036. organizational factor (aOR: 0.087 CI (0.034–0.22), *P* = 0.000 individual beliefs factor (aOR: 0.19 CI (0.07–0.48), *P* = 0.000 and attitudes (aOR: .22 CI (0.08–0.61), *P* = 0.004. Based on the qualitative findings, the main obstacles to home-based maternity and child health care were found to be infrastructures, finances, and implement burden.

**Conclusion and recommendation:**

This study found that many midwives were willing to offer child health care and home-based maternity services. Establishing and implementing a home-based maternity and child healthcare service requires sufficient manpower, facility equipment, and access infrastructure.

## Introduction

The World Health Organization (WHO) defines maternity and child health care services as essential components of the continuum of care for mothers and newborns, aimed at improving their well-being before conception and extending up to six weeks post-delivery (1, 2). This period, known as the “maternity and child health care late period,” is critical for the health and survival of both mother and child. While 71% of women and 64% of newborns receive customary postpartum care within two days of delivery, significant gaps remain in adequate support during this time (3). Funding midwife-led interventions could prevent approximately 2.5 million neonatal deaths annually, as neonatal fatalities account for about 70% of newborn mortality in low- and middle-income countries (4, 5). Adequate postnatal care (PNC), which includes health assessments, vaccinations, and breastfeeding education, is essential to address this issue.

The Sustainable Development Goals (6) target 3.1 aims to reduce global maternal mortality rates to less than 70 per 100,000 live births by 2030 (6, 7). However, achieving this goal is challenging in African countries like Ethiopia, where maternity care, particularly postnatal care, remains insufficient. Ethiopia's Second Health Sector Transformation Plan (HSTP II) aims to lower neonatal mortality rates from 33 to 21 per 1,000 live births and maternal mortality rates from 401 to 279 per 100,000 live births by 2024–2025 (8). To meet these objectives, a strong focus on maternal and child health care is required, as up to 65% of maternal deaths are attributed to postpartum complications (9). Infants are particularly vulnerable in the first month after delivery, with a global average neonatal mortality rate of 17 deaths per 1,000 live births in 2019, and even more in Ethiopia (10). Furthermore, 64% of women in Ethiopia did not receive a maternity and child health care check, and only 2% received care from a health extension worker as 2019 EDHS (Ethiopian Demographic Health Survey) reports. The inconsistent implementation of care by midwives at home further exacerbates these issues. Evidence suggests that maternity and child health care (MCHC) programs, especially postnatal care, are among the least effective reproductive and child health services. The WHO recommends that midwives visit women at home at least once to enhance their knowledge of maternal and newborn health (11).

In Africa, including Ethiopia, experience with home-based maternity and child health care (HB-MCHC) is limited, although several studies are exploring adapted approaches that connect this care to the health system (12). As hospital stays post-delivery have decreased, various factors, including the risk of infections and stress (13, 14), underscore the importance of implementing HB-MCHC. Women in Ethiopia face numerous challenges in accessing maternity and child health care, including poverty, geographical barriers, lack of information, and cultural restrictions on movement during the early postpartum period (15, 16). Implementing home-based care can effectively improve accessibility and ensure timely support. To reduce maternal and neonatal mortality and morbidity, midwives can provide HB-MCHC services in women's homes after discharge from health institutions. This study aims to assess the willingness of midwives to implement HB-MCHC and identify the barriers they encounter in doing so. It seeks to answer the following questions: What factors influence midwives' willingness to deliver HB-MCHC in Gondar, and what barriers do they face in implementing these services?

### Conceptual framework

## Methods

### Study design

This study employed an institutional-based mixed-methods design. Data collection for the quantitative phase occurred from January 23 to February 23, 2023, in public health facilities in Gondar, Ethiopia.

### Study participants

The study targeted midwives working in central Gondar zone public health facilities. The midwives included in the quantitative phase were selected regardless of their experience level to provide a broad perspective on willingness to offer home-based maternity and child health care (HB-MCHC). Geographically, Gondar is located in the Northwestern Highlands of Ethiopia's Amhara Regional State, approximately at latitudes 12°36′ N and 37°28′ E. It is about 120 kilometers from Bahir Dar, the capital of the Amhara National Regional State, and 727 km from Addis Ababa, the capital of Ethiopia (17). The population of Gondar town and central Gondar is approximately 3 million. According to local administrators, there are around 471 midwives' working in Central Gondar and the public health institutions, which include hospitals and clinics in the central Gondar zone. Gondar town is home to the University of Gondar Comprehensive Specialized Referral Hospital, the only specialty referral hospital in the area. Central Gondar has nine primary hospitals—Denbya, Delgi, Shawera, Aykel, Tsegede, Sanja, Guhala, Arbaya, and Ambagiorgis—and 76 health centers.

### Data processing and quantitative data analysis

For the quantitative research, all data were manually verified for completeness, coded, and input into the Epi-Data version 4.6 program. The data were then transferred to SPSS version 21 for analysis. Descriptive statistics, including frequencies, percentages, and median with interquartile range (IQR), were used to summarize the data. Analytical and descriptive statistical techniques facilitated the execution of binary logistic regression to identify significant relationships between independent and dependent variables. Each independent variable's relationship with the outcome variable was examined using binary logistic regression.

To be included in the multivariable logistic regression model, a variable had to have a *p*-value of less than 0.25. Subsequently, statistically significant correlations were assessed using the adjusted odds ratio (aOR), along with its 95% confidence interval and a *p*-value of less than 0.05 (18). The findings were compiled and presented through text, tables, and graphs, with a focus on reviewing the odds ratios and their corresponding confidence intervals.

### Inclusion and exclusion criteria

**Inclusion Criteria:** All fully employed midwives at public health facilities in Gondar and the central Gondar zone;

**Exclusion Criteria:** Midwives who were on annual vacation or any other leave during the data collection period.

### Quantitative data collection processes and tools

A pre-tested, semi-structured, self-administered questionnaire was developed for the quantitative phase, drawing on relevant literature to ensure content validity. The instrument contained five sections: sociodemographic characteristics, organizational factors, willingness to implement home-based maternity and child health care (HB-MCHC), individual beliefs, and social support. Its purpose was to capture comprehensive information on midwives' perspectives regarding HB-MCHC. To enhance relevance, the questionnaire incorporated rating scales and items aligned with the International Confederation of Midwives (ICM) performance requirements, thereby allowing responses to be assessed against internationally recognized benchmarks in maternity and child health care (19).

Data collection was conducted using the pretested questionnaires, which were distributed by six midwives with BSc degrees recruited as supervisors. The process was coordinated and overseen by the principal investigator. A pretest involving 22 midwives (5% of the total sample) was carried out to evaluate internal consistency and completeness of the items, yielding a Cronbach's alpha of 0.772, which indicated acceptable reliability. To further ensure data quality, the lead investigator and supervisors routinely checked completed questionnaires for accuracy and completeness.

### Data quality control

To ensure data integrity, supervisors and data collectors received two days of training on the study objectives and data collection procedures. During the fieldwork, data collectors operated under continuous supervision to maintain consistency. The instruments underwent expert review to establish face validity. A pretest was conducted to evaluate internal consistency and completeness of the questionnaire items, and reliability was assessed using Cronbach's alpha in SPSS version 21. The resulting coefficient of 0.772 indicated acceptable reliability of the instrument (20, 21).

### Sample size and sampling procedures

The sample size was determined by using single population proportion formula using 95% confidence level (*Z* = 1.96), degree of precision (marginal error = 5%), and proportion $\left(\;p=50\text{\%}\;n=\frac{z^{2}*p(1-p)}{d^{2}}\right)$ where;

*n* = the required sample size
*P* = proportion of midwives'; 50% = 0.5 (since there was no previous study in Ethiopia)
*z* = degree of accuracy at 95% = 1.96
*d* = margin of erro*r* = 0.05
  $$n=\frac{{\left(1.96\right)}^{2}\;*\;0.5\;(1-0.5)}{{\left(0.05\right)}^{2}}=384$$

None response 38.4 ≈ 39 (10%). The final sample size was 423.

### Operational definitions

**Willingness:** A mental state reflecting a commitment to providing home-based maternity and child health care (HB-MCHC) in the future (22).

**High level of willingness**: Willingness was assessed using a Likert scale composed of seven items. A composite score was calculated, with a mean of 13 and no outliers detected. A score of ≥13 was classified as a high level of willingness to implement HB-MCHC.

**Level of midwives' attitude**: Refers to the attitudinal items related to willingness to implement HB-MCHC (23). The total score was derived from four items, with a possible range up to 27.

**Positive attitude:** Scores above the mean (≥7) indicated a positive attitude toward HB-MCHC.

**Organizational support:** Defined as the support and resources provided by health facilities to midwives. Eight items were evaluated, with the mean score calculated after confirming no outliers.

**High organizational support:** Scores above the mean (≥8) were classified as high organizational support.

**Individual beliefs**: Refers to personal convictions, values, and opinions that may influence midwives' behavior, decision-making, and interactions (24).

**Positive individual beliefs**: Midwives scoring above the mean (≥11) were considered to hold positive individual beliefs.

**Social support**: Defined as the assistance midwives receive from family, friends, colleagues, and community members. This construct was measured using five items.

**High social support**: Scores above the mean (≥9) indicated a high level of social support.

### Study variables

The dependent variable is the willingness to implement home-based maternity and child health care (HB-MCHC), indicated as Yes/No. Independent variables include socio-demographic factors such as age in years (24–29, 30–34, 35–39, ≥40), sex, midwives' residence, marital status, educational status, work experience, family loss history related to obstetrics, monthly income [<5,000 birr (<40$), 5,000–8,000 birr (40–62$), >8,000 birr (>62$)], working facility, midwife's family residence, and midwives' number of children. Additionally, the level of organizational support factors (high or low), encompasses the availability of MCHC rooms, midwives' previous experience with HB-MCHC, working department, team dynamics, organizational support, and availability of organizational MCHC services.

The age categories were defined based on a review of existing literature on midwife demographics and workforce participation (25, 26). These categories also reflect typical career stages, where midwives in the 24–29 age group are likely to be early in their careers, those in the 30–39 age range represent mid-career professionals, and those ≥40 may have more extensive experience. These distinctions can influence their willingness to adopt new practices such as HB-MCHC.

Justification for income categories: The income categories were determined using current data from administration staff regarding midwives' salary scales within the study region. These categories are designed to reflect the economic conditions and standards of living for midwives in the specific context. The cutoffs at <5,000 Birr, 5,000–8,000 Birr, and >8,000 Birr, correspond to different levels of financial stability and access to resources, which may impact their willingness to implement HB-MCHC. Furthermore, the level of individual beliefs factors (positive or negative), includes midwives' confidence, extra payment incentives, perceived risks associated with HB-MCHC services, motivation, and job satisfaction.

**Figure 1:**
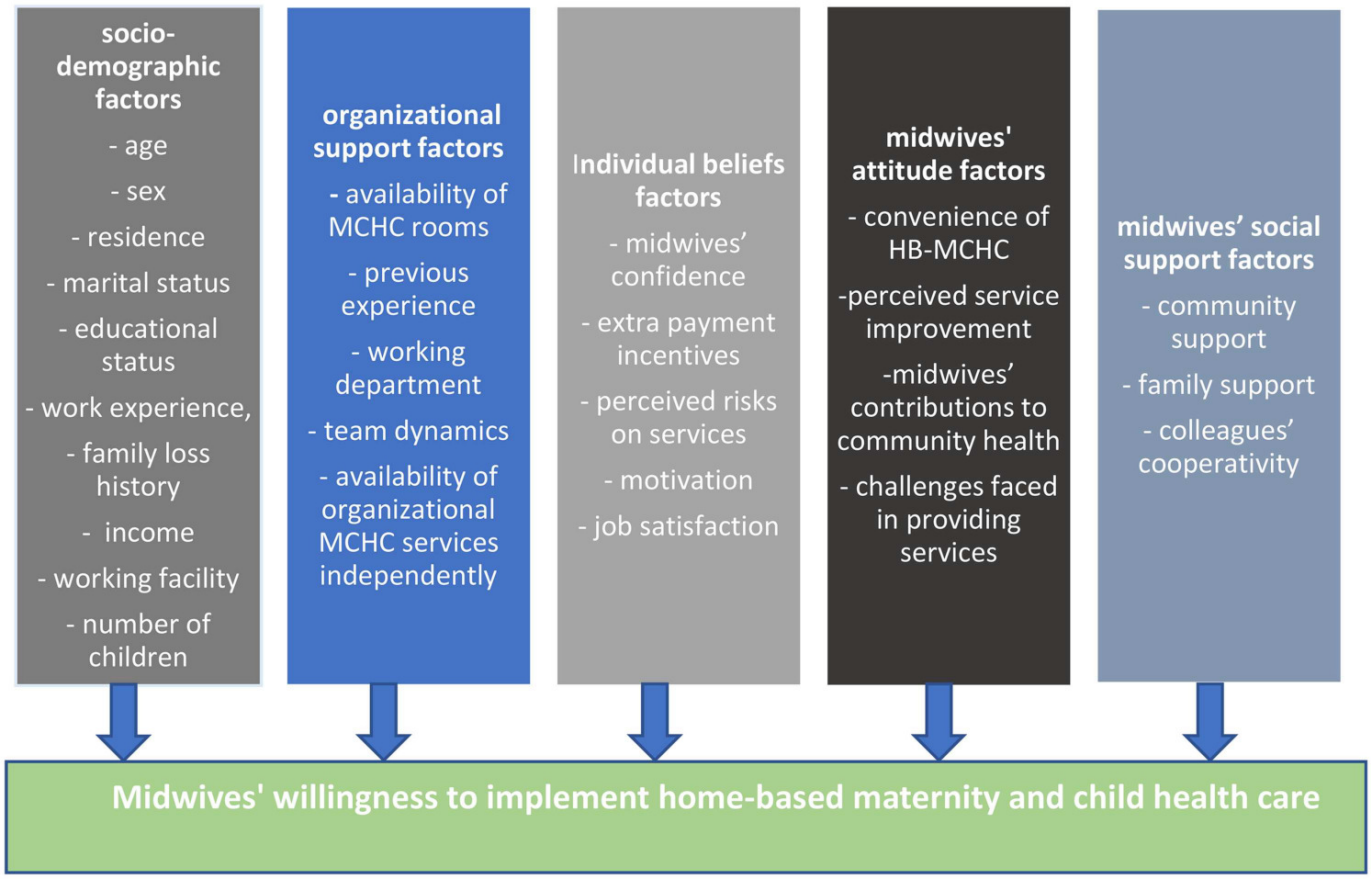
Conceptual framework of Midwives' willingness to implement home-based maternity and child health care.

The level of midwives' attitude factors (positive or negative), consists of the convenience of HB-MCHC, perceived service improvement, midwives' contributions to community health, and challenges faced in providing services. Lastly, the level of midwives' social support factors (high or low), involves community support, family support, and colleagues' cooperativity, (Figure 1).

### Qualitative data analysis

#### Purpose of the qualitative phase

The qualitative phase of this study aimed to gain in-depth insights into the barriers and facilitators influencing midwives' willingness to implement home-based maternity and child health care (HB-MCHC). While the quantitative data provided a broad overview of willingness, qualitative interviews allowed for a deeper exploration of the underlying factors affecting midwives' perceptions and experiences.

#### Data collection processes and tools

To ensure relevance, the interview questions for the qualitative phase were developed following a thorough analysis of the quantitative findings. Experts in midwifery and public health crafted these questions to probe deeper into the factors that encourage or hinder midwives' willingness to provide HB-MCHC. Qualitative interviews were conducted concurrently with the quantitative phase. The lead investigator performed face-to-face in-depth interviews with twelve midwives, each with more than three years of experience. This selection criterion was intended to ensure that participants possessed sufficient knowledge and insights regarding the issues influencing their willingness to implement HB-MCHC. Data collection concluded after reaching saturation, meaning no new information emerged from the interviews.

MCHC team leaders at each study health institution assisted in identifying potential research participants, who were contacted and informed about the study. Participants received an invitation letter detailing the time and date of the interview. To facilitate effective discussions, interviews were conducted at the participants' workplaces. Digital devices captured audio recordings of the interviews, and supplementary notes were taken to enhance the richness of the data.

#### Data preparation

Audio recordings were transcribed verbatim in Amharic and subsequently translated into English by a bilingual expert to preserve linguistic nuance. Transcripts were checked for accuracy by comparing them against the original recordings. The translated transcripts were formatted into Microsoft Word files and imported into ATLAS.ti software for systematic coding and analysis.

#### Analytical approach

The qualitative data were analyzed using thematic analysis, guided by Braun and Clarke's six-phase framework. The researcher first immersed themselves in the transcripts through repeated readings to gain a holistic understanding of participants' perspectives. Initial codes were then generated by identifying meaningful segments of text, which were systematically organized using ATLAS.ti software (27, 28).

These codes were subsequently clustered into categories that reflected conceptual similarities, allowing the researcher to search for and refine emerging themes. The themes were reviewed against the raw data to ensure consistency and accuracy, with discrepant cases examined to sharpen thematic boundaries. Finally, each theme was clearly defined and named, and the findings were reported through narrative descriptions supported by illustrative quotations from participants. This structured approach ensured that the analysis captured both explicit statements and underlying meanings, thereby providing a comprehensive account of the barriers and facilitators influencing midwives' willingness to implement HB-MCHC (29).

## Trustworthiness

To enhance the credibility and rigor of the qualitative findings, several strategies were employed throughout the analytic process. Triangulation was achieved by comparing qualitative themes with quantitative results to identify areas of convergence and divergence. Peer debriefing was conducted with qualitative research experts, who reviewed coding decisions and thematic structures to strengthen dependability (30, 31). Member checking was used to validate interpretations, as summaries of findings were shared with a subset of participants to confirm accuracy. An audit trail was maintained to document coding decisions, analytic memos, and reflections, thereby ensuring transparency and confirmability. Collectively, these measures reinforced the trustworthiness of the analysis and ensured that the findings authentically represented the perspectives and experiences of the participating midwives.

## Result

### Socio-demographic characteristics of the respondents

The initial study included 423 midwives, yielding an impressive response rate of 98.3% (416 participants). Among those surveyed, women constituted nearly two-thirds (62.5%) of the sample. Approximately 63% of participants were married, with around 40% falling within the 30- to 34-year age range. A significant majority of respondents (81%) had over three years of work experience and held a bachelor's degree. Monthly income for about 85% of participants ranged from 5,000 to 8,000 birr (40–62$). Notably, nearly two-thirds (58.4%) of the study participants reported not having children, and almost half (48.3%) were employed in health centers. Furthermore, more than half of the participants (53.8%) lived in rural areas. A history of family loss due to childbirth was reported by nearly 25% of the respondents (Table 1).

**Table 1:** Sociodemographic characteristics of midwives' participants.

Variables	Categories	Frequency (*N* = 423)	Percentage (%)
Sex	Male	156	37.50%
	Female	260	62.50%
Age in Years	24–29	123	29.60%
	30–34	167	40.10%
	35–39	93	22.40%
	≥40	33	7.90%
Midwives' Residences	Urban	237	57.00%
	Rural	179	43.00%
Marital status	Single	120	28.80%
	Married	262	63.00%
	others	34	8.20%
Educational status	Diploma	54	13.00%
	BSc	338	81.30%
	MSc	24	5.80%
Work experience	≤1 year	25	6.00%
	>1–<3	317	76.20%
	≥3 year	74	17.80%
Monthly income	<5,000 birr (<40$)	33	7.90%
	5,000–8,000 birr (40–62$)	353	84.90%
	>8,000 birr (>62$)	30	7.20%
Participants work area	Referral Hospital	160	38.50%
	Primary hospital	55	13.20%
	Health center	201	48.30%
Family Residence	Urban	192	46.20%
	Rural	224	53.80%
Having Children	Have no children	243	58.40%
	≥1 child	173	41.60%
Midwives' Religion	Orthodox	326	78.40%
	Muslim	78	18.80%
	Others	12	2.90%
Midwives' Ethnicity	Amhara	386	92.80%
	Others	30	7.20%
Obstetrics related family loss	Yes	116	27.90%
	No	300	72.10%

HB-MCHC, home-based maternity and child health care.

### Results of midwives' willingness to implement HB-MCHC

The study concluded that among the 416 midwives' surveyed, nearly three out of four (74.3%, with a 95% confidence interval of 74%–77.4%) expressed a desire to Implement home-based maternity and child health care (HB-MCHC). However, only 9% reported having prior experience with HB-MCHC services. Additionally, nearly 85% of participants were involved in postnatal care (PNC), antenatal care, and labor and delivery departments.

**Figure 2:**
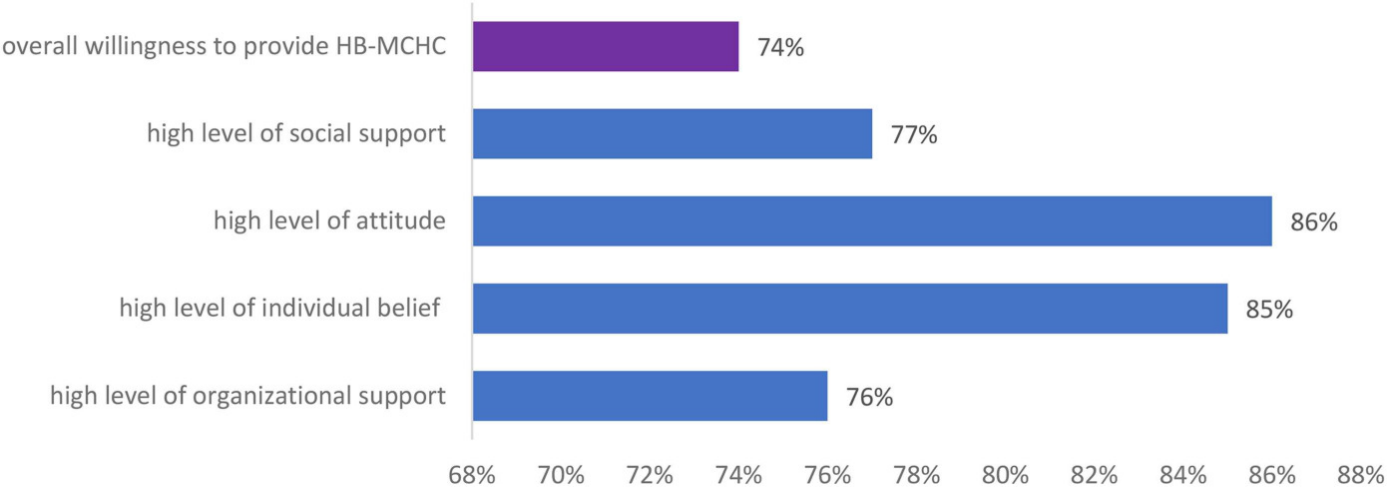
Midwives' willingness to implement HB-MCHC and factors, in Gondar (HB-MCHC: home-based maternity and child health care).

**Figure 3:**
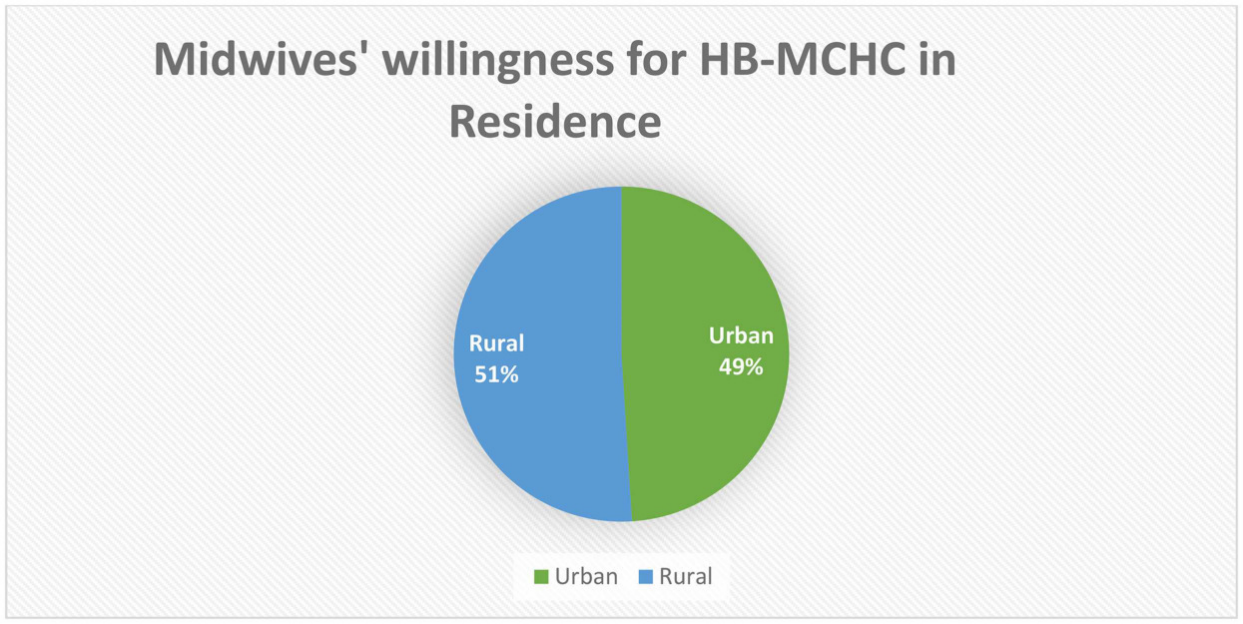
Willingness for HB-MCHC (home-based maternal and child health care) in residence.

More than two-thirds (76%) of the 416 midwives' who participated in this study demonstrated a strong desire to offer HB-MCHC, with about 84% responding to the eight organizational support questions. A total of three hundred fifty-three participants (85%) expressed a commitment to providing HB-MCHC, driven by strong personal convictions, while 359 respondents (86%) held favorable opinions about delivering these services. Notably, 321 midwives' (77%) reported having a high level of social support for providing HB-MCHC (Figure 2), and 158 midwives' (51%) living in rural areas were willing to Implement HB-MCHC (Figure 3).

In the binary logistic regression analysis, a *p*-value of 0.25 revealed a significant correlation between eleven out of seventeen variables and the midwives' readiness to offer HB-MCHC. However, after applying multivariable logistic regression to account for potential confounding factors, significant variables included experience of obstetrics-related family loss (aOR: 0.19, CI: 0.07–0.48), organizational factors (aOR: 0.87, CI: 0.034–0.22), and midwives' attitudes (aOR: 0.22, CI: 0.08–0.61). A high level of desire to Implement HB-MCHC was significantly correlated with an adjusted odd ratio (aOR) of 2.2 (CI: 1.04–4.8) (Table 2).

**Table 2:** Bi-variable and multivariable analysis of factors associated with the willingness of midwives to provide HB-MCHC.

Variables	Category	Willingness of midwives' to HB-MCHC		cOR (95% CI)	*P*-value	aOR (95%)	*P*-value
		Yes	No				
Age	24–29	84	38	0.47 (0.25–0.85)	0.13	1.35 (0.46–3.95)	0.58
	30–34	121	47	0.54 (0.31–0.96)	0.37	0.93 (0.37–2.34)	0.87
	≥35	104	22	1			
Gender	Male	110	46	.73 (.47–1.14)	.174	1.1 (.58–2.1)	0.77
	Female	199	61	1			
Participant education	Diploma	44	10	2.64 (0.9–7.7)	0.225	0.99 (0.29–4.5)	0.99
	BSc	250	88	1.7 (0.72–4.03)	0.077	1.2 (0.33–4.37)	0.76
	MSc	15	9				
Work experience	≤1 year	17	8	0.37 (0.13–1.06)	0.066	0.96 (0.19–4.84)	0.964
	>1–<3	229	88	0.45 (0.23–0.90)	0.024	0.81 (0.24–2.69)	0.728
	≥3 year	63	11	1			
Midwives' has Children	Have no	167	76	0.48 (0.29–0.77)	0.002	0.93 (0.45–1.88)	0.838
	≥1	142	31	1			
Family residence	Urban	151	41	1			
	Rural	158	66	0.65 (0.41–1.02)	0.060	0.68 (0.37–1.25)	0.218
Obstetrics Family loss History	Yes	100	16	2.72 (1.5–4.8)	0.001	2.2 (1.04–4.8)	**0** **.** **036** *
	No	209	91	1			
Level of midwives' organizational support Factors	High	295	60	16.5 (8.5–31.8)	0.000	0.087 (0.034–0.22)	0.000*
	Low	14	47	1			
Level of midwives' Beliefs Factors	High	296	57	19.9 (10.2–39.1)	0.000	0.19 (0.07–0.48)	0.000*
	Low	13	50	1			
Level of midwives' Attitude Factors	High	297	62	17.96 (8.98–35.9)	0.000	0.22 (0.08–0.61)	0.004*
	Low	12	45	1			
Level of midwives' Social support Factors	High	276	45	11.52 (6.8–19.5)	0.000	0.5 (0.23–1.1)	0.085
	Low	33	62	1			

Significant variables are indicated in bold, with asterisks indicates associated factors significant at *p* < 0.05.

HB-MCHC, home-based maternity and child health care.

aOR, adjusted Odds Ratio.

### Barriers to midwives' willingness to implement HB-MCHC

1. **Workload-related factors:** Participants highlighted that the workload at health facilities and the busyness of healthcare providers significantly hinder the provision of home-based maternity and child health care (HB-MCHC). One midwife noted, “ I don't think there is enough time for HB-MCHC with this number of midwives'. With a limited number of colleagues, it's difficult to implement this; however, if there are many midwives' assigned for this purpose, it will be possible to address HB-MCHC properly.” *(Midwife 5)*
  Inadequate health provider staffing affects service delivery, while high patient case flow strains available resources (32, 33). Additionally, time-consuming procedures further limit the time available for home visits.
2. **Infrastructure-related factors**: Midwives reported that inadequate infrastructure significantly impacts their willingness to offer HB-MCHC. Key issues include a lack of medical equipment and transportation. One midwife stated, “Many times, challenges arise due to various reasons, such as transportation issues related to infrastructure and a scarcity of medical equipment.” *(Midwife 1)*
  The dispersed nature of rural homes, combined with poor transportation and road conditions, further complicates their ability to deliver services. “On one occasion, there was an emergent obstetric event where a mother had bleeding before labor. We couldn't find a vehicle or ambulance immediately. It took more than two hours, and we almost lost her.” *(Midwife 6)*
  Transportation issues make it difficult to access patients in remote areas, while a lack of medical equipment results in insufficient resources to implement care (34, 35). Additionally, safety and security concerns pose risks associated with traveling to patients' homes.
3. **Financial factors**: Financial constraints are a critical barrier affecting the provision of HB-MCHC. Many midwives' reported low income and a lack of additional incentives as significant issues. “It is challenging to Implement services like HB-MCHC without additional funding or incentives because it requires extra work and time, which cannot be covered out of pocket.” *(Midwife 2)*
  Midwives' expressed a need for better financial support to enhance their willingness to implement home-based services. “I don't think functional HB-MCHC provision is possible in our setup unless extra payment is allowed.” *(Midwife 3)*
  Additionally, insufficient collaboration and communication among midwives and stakeholders were noted. “It would be beneficial if there were programs or guidelines to facilitate communication and collaboration” *(Midwife 8).*
  The high demand for services, with an increasing number of mothers and babies in need of care, adds pressure on midwives. “Sometimes it is challenging to deliver MCHC to those who require it but live too far from a medical center” *(Midwife 6).*

The barriers identified underscore the need for systemic changes to enhance the provision of HB-MCHC. Addressing workload, infrastructure, and financial challenges will be crucial in improving service delivery (36, 37), and midwives' willingness to implement care.

### Facilitators to midwives' willingness to implement HB-MCHC

The key theme identified as a facilitator for midwives in providing HB-MCHC mirrored the barriers, focusing on workload-related factors, financial support, and infrastructure resolution.

1. **Organizational Support**: Many midwives emphasized the importance of organizational support in enhancing their willingness to implement home-based care. Over 90% reported that suitable staffing levels contributed to their ability to deliver quality care. “If there are enough midwives to meet the demand for HB-MCHC services, it can ensure that we are not overworked and can implement high-quality care for our clients” *(Midwife 5).* Access to essential supplies and equipment, such as emergency kits, blood pressure monitors, and neonatal resuscitation tools, is crucial for midwives to deliver safe and effective care (38, 39). “We would like to implement HB-MCHC when there is adequate infrastructure and logistics. With the collaboration of other stakeholders, all midwives will be willing to provide this service” *(Midwife 3).*
2. **Continuing Education and Professional Growth**: The majority of midwives highlighted the importance of continuing education and professional development. Ongoing training and support supervision are vital for building their confidence and competence in providing HB-MCHC. Regular monitoring and feedback from supervisors enable midwives to identify areas for improvement and enhance their clinical skills. “With the support of regular monitoring and feedback, we gain confidence in our skills and can better identify areas for growth” (*Midwife 7*).

Overall, providing organizational support to midwives can significantly improve the quality of care they deliver. By addressing workload, ensuring access to necessary resources, and facilitating ongoing education (40), healthcare systems can enhance midwives' willingness to implement high-quality HB-MCHC, ultimately benefiting the health of expectant women and their babies.

## Discussion

This research aimed to assess midwives' willingness to provide home-based maternity and child health care (HB-MCHC) in the Gondar region, identify strategies to enhance MCHC services, and address inadequate service provision. Engaging with mothers and newborns during postpartum visits is essential, as midwives' play a crucial role in delivering care. Understanding the risks and commitments associated with their profession is vital, given that midwives' are the primary providers of HB-MCHC. To improve MCHC delivery and reduce maternal and infant mortality, it is essential to evaluate midwives' willingness to participate in these services.

This study utilized both qualitative and quantitative mixed methods to investigate midwives' willingness to implement HB-MCHC in Gondar town. The findings indicated that 74.3% of midwives were willing to offer HB-MCHC, with a 95% confidence interval (CI) of 74%–77.4%. This willingness aligns with a study conducted in Canada, which reported a similar figure of 77.1% (41). The comparable study populations and large sample sizes may explain these similarities. However, the proportion in this study is lower than that found in a prior study in Sri Lanka, where 83% of midwives' expressed willingness (42). Differences in socioeconomic status and the state of the healthcare system could account for this discrepancy. Additionally, prior experience with HB-MCHC may vary, as the service is more commonly established in rural areas, where populations might be more familiar with such offerings (43).

In comparison to other studies, the current findings show a higher willingness than studies conducted in Ethiopia (59.5%) (44), Nigeria (42%) (23) and Netherlands 57% (45). The lower willingness observed in the Ethiopian study may be attributed to the pandemic's impact on healthcare delivery and the limited number of health facilities involved. Additionally, the study in Nigeria had a smaller sample size and fewer facilities, which could influence results.

In this study, level of organizational support factors, level of midwives' beliefs factors, level of midwives' attitude factors and obstetrics family loss history are associated factors which are significant to willingness implement to HB-MCHC.

A notable finding in our study is that midwives' with high levels of belief in the importance of HB-MCHC had an 81% higher likelihood of being willing to implement these services compared to those with lower belief levels. This finding is consistent with prior research in Nigeria, which also highlighted a strong association between midwives' beliefs and their willingness to offer home-based services (23). Furthermore, qualitative data from our study indicated that midwives' who received high organizational support were more inclined to plan for providing HB-MCHC.

In contrast, other research has indicated that in African nations, there is a strong correlation between inadequate staffing and excessive overtime, which can further complicate service provision (46). Addressing these challenges through organizational support and resources will be crucial in enhancing midwifes' willingness to implement HB-MCHC and ultimately improving maternal and newborn health outcomes. The current study's findings align with previous research, revealing a significant relationship between high levels of willingness to implement HB-MCHC and elements of organizational support (23).

This study identifies significant factors associated with midwives' willingness to implement HB-MCHC, including a history of obstetric family loss, strong organizational support, positive beliefs about HB-MCHC, and a favorable attitude towards providing care.

Midwives' with strong individual beliefs were approximately 3.66 times more likely to demonstrate a high willingness to implement HB-MCHC, a finding consistent with prior studies (44). This may indicate that midwives' who work as a cohesive team tend to share similar perspectives and attitudes towards providing woman-centered care (47).

Furthermore, the study found that midwives with a high level of positive attitude had a 78% higher likelihood of being willing to implement HB-MCHC compared to those with a lower level of attitude. Specifically, midwives' exhibiting a strong attitude were nearly five times more likely to intend to implement these services. This finding is further supported with other studies (48–50), and by qualitative insights, with one midwife stating: “When I believe in the importance of home-based care, I feel more motivated to provide it, knowing that it can make a real difference in the lives of mothers and their newborns.” This underscores the need for training and support that fosters positive attitudes among midwives, as their willingness to provide care can significantly impact maternal and neonatal health outcomes. Overall, these results suggest that enhancing midwives' perceptions of the value of HB-MCHC could be an effective strategy in addressing maternal and newborn mortality rates, particularly in resource-limited settings.

## Conclusion and recommendations

This study found that midwives' willingness to provide home-based maternity and child health care (HB-MCHC) is generally adequate, influenced by personal histories, organizational factors, attitudes, and beliefs. However, barriers such as inadequate infrastructure, high workloads, and financial constraints were noted. To enhance HB-MCHC provision, it is crucial to improve infrastructure by investing in transportation and communication systems. Reducing workloads through better staffing and resource allocation is essential, alongside providing financial support like incentives. Implementing a mixed strategy that integrates facility-based care with home-based services for mothers and children will ensure comprehensive care continuity. Additionally, equipping midwives with the necessary tools and resources for effective home care is vital.

Ongoing training and professional development will empower midwives, while fostering a positive organizational culture will ensure they feel valued. Addressing individual beliefs through workshops can help overcome misconceptions. Future research should evaluate the effectiveness of these strategies in empowering midwives and improving the quality of home-based care, ultimately enhancing maternal and child health outcomes.

## Data Availability

The original contributions presented in the study are included in the article/Supplementary Material, further inquiries can be directed to the corresponding author.
